# Dental, Oral and General Health of Geriatric In-Hospital Patients Before Immediate Prosthetic Treatment: A Retrospective Cohort Study

**DOI:** 10.3390/dj13080334

**Published:** 2025-07-22

**Authors:** Michael Pampel, Jana Kraft, Thomas Tümena, Johannes W. Kraft

**Affiliations:** 1 TMD-Center, Private Practice, Sana Hospital Coburg, 96450 Coburg, Germany; 2 University Postgraduate Study Program Evidence Based Medicine, School of Medicine, University of Split, 21000 Split, Croatia; 3 Faculty of Social Work and Health, University of Applied Sciences, 96450 Coburg, Germany; jkraft-laurentius@web.de (J.K.); johannes.kraft@regiomed-kliniken.de (J.W.K.); 4 Medical Society for the Promotion of Geriatrics in Bavaria (Geridoc), 95444 Bayreuth, Germany; thomas.tuemena@geridoc.de; 5 Geriatrics in Bavaria Database (GiB-DAT), 90475 Nuremberg, Germany; 6 Rehabilitation Department, Sana Hospital, 96450 Coburg, Germany

**Keywords:** geriatrics, dentistry, in-hospital, morbidity, intraoral risk factors, masticatory function, malnutrition, databank

## Abstract

**Objectives**: The relationship between oral health and general health of geriatric in-hospital patients (GIH) who are poly-morbid and edentulous is currently unclear. This study determined the relationship between oral health and general health, and further implications and recommendations were derived. **Methods**: This retrospective cohort study included 81 GIH patients with impairment of oral state and masticatory function and need for immediate prosthetic treatment. The number of medical diagnoses, particularly main diagnoses of being hospitalized, comorbid diagnoses and the dental/oral state, were evaluated. Laboratory data of vitamin D3 and albumin concentrations were measured. Intraoral risk factors (IRF) affecting the masticatory function were intraoral inflammation, mucogingival impairment (MGI) and severe bone crest atrophy (SBCA). Masticatory function was evaluated by DMF*-T Index (number of destroyed/diseased, missing teeth and artificial fabrication), Eichner Index and Scores. The clinical relevance was surveyed by significance and effect size calculations. **Results**: In GIH, the number of medical diagnoses correlated significantly with the occurrence of IRFs. SBCA was the most affecting IRF, as measured by Eichner Index at baseline (*p* = 0.001). Single main diagnoses CNS and gastro-intestinal disease (GID) correlated with both deficiency of vitamin D3 levels (*p* = 0.011; *p* = 0.028) and hypoalbuminemia (*p* = 0.013; *p* = 0.023). Single comorbid diagnoses significantly correlated with both vitamin D3 deficiency and hypoalbuminemia (CVD (*p* = 0.031); DM (*p* = 0.042). Hypoalbuminemia was further found to be correlated with the sum of comorbid diagnoses (*p* = 0.033). **Conclusions**: GIH patients suffered from general and dental poly-morbidity. The prevalence of diseases was higher due to SBCA and impaired masticatory function. Deficiency of vitamin D3 and hypoalbuminemia were possible malnutrition markers.

## 1. Introduction

As reported by the WHO Global Oral Health Data Bank “poor oral health during old age is mostly manifest in […] severe tooth loss” [1]. “ Impaired mastication and nutrition intake, which relate to general health, are strongly affected by an individual’s dental status” [2]. However, the link between dental status and general health is frequently underestimated and a combined approach by physicians and dentists is typically considered as not achievable in clinical routine, especially for elderly and frail patients of geriatric units. Previous studies have already established relations between dental health and general health. As found in the German oral health survey 2016 [2], tooth loss and eventually edentulism constitute the final stage of caries and periodontal disease. In Germany, edentulism is a problem of the elderly, especially in the age group of 75 to 100 years, where 32% suffer from total edentulism [2]. Although losing teeth is still seen as a natural consequence of ageing by many [3], it has been demonstrated that edentulism relates to high prevalence of comorbidities [4,5,6] and may be an important predictor of mortality [7]. When edentulism appears, high prevalence rates for comorbidities, such as hypertension, diabetes mellitus, and chronic depression or psychiatric disease in general, exist [4,5]. The problem of edentulism is increased when masticatory function is reduced, as removable prostheses are not as efficient as natural dentition or fixed dentures [8]. The number of natural teeth and occlusal units is also associated with masticatory function, which can be measured by objective methods [9,10,11] since subjective methods [12,13] are influenced by self-perception bias [14]. A number of natural teeth less than ten is associated with reduced masticatory ability; the presence of three to four premolar pairs is most important [15]. Missing teeth are the strongest influential factor for nutrition [16].

Dental and oral health findings are further important factors for malnutrition, which in turn is a risk factor for general morbidity and mortality [17,18]. In fact, a significant relationship between nutrition and loose fit of dentures has been found in previous research [19]. Recently, a relation between edentulism and protein-energy-related malnutrition was evidenced [20]. Tooth loss is also considered as a risk factor for dementia [21,22] and for mild cognitive impairment (MCI) [23]. The existing dental and general medical data have previously not been sufficiently implemented in daily hospital routines and did not lead to practical implications [15,17]. Due to a gap in guideline recommendations and the economical urge to maintain valuable and low costs by dental care in hospital departments, it becomes clear that there is a lack of cooperation between hospital staff and dentists. To reduce this gap, the present paper aims to answer the following research questions: What is the relationship between dental/oral and general health medical findings in geriatric in-hospital patients? Can dysfunctional, ill-fitting dentures and missing teeth of GIH patients cause general health deterioration?

## 2. Materials and Methods

### 2.1. Study Design

The retrospective cohort study was executed. The Ethics Committee of the University of Applied Sciences Coburg (HC_Kraft_Pampel_29082018) and the Institutional Review Board of the Regiomed Medical School Coburg (STWAMICA) approved the study protocol.

### 2.2. Participants and Sampling Methods

A priori power analysis was conducted to determine the required sample size for a two-tailed paired *t*-test comparing dependent means with sample size calculator G*-Power (version 3.194). Assuming a small-to-medium effect size (dz = 0.3), a significance level of α = 0.05 and a desired power of 0.8, the analysis indicated a total sample size of 90 participants.

For this study, 110 geriatric patients were consecutively recruited from the in-patient rehabilitation department at the hospital in Coburg, Germany, from 01/2015 to 06/2017. Before admission, they were in severe condition in the acute hospital. The study group was comprised of patients (age > 60 years) hospitalized for rehabilitation (GIH) and in need of immediate prosthetic treatment (IPT) due to signs of malnutrition, pathological dental/oral findings or intraoral complaints. Preliminary patient selection was executed by the treating medical doctors, and this baseline data collection started before dental treatment. Patients with disease settings directly interfering with masticatory function, such as palliative stage cancer of digestive organs, head or neck, and patients after surgery of these areas were excluded from the analysis (n = 6). Exclusion criteria and drop-outs due to deterioration of health and cognition resulted in a final study group of 81 patients as the convenience sample. Most of the GIH patients stayed at the acute hospital for diagnosis, therapy and operations before rehabilitation and respectively went on to intensive care units, intermediate care units and early rehabilitation wards as necessary. This was usually followed by admission to expansive in-hospital geriatric rehabilitation. Patients had an average stay of three weeks in rehabilitation, in which the observations for this study were collected. In sum, the duration of hospitalization emphasizes the high morbidity of the GIH cohort.

To yield more representative results, the baseline data obtained from this study’s single-center cohort were compared with a large multi-center databank (Geriatrics in Bavaria, GiB-DAT) of around 36,500 geriatric patients per year from 56 geriatric departments in Bavaria, Germany [24,25], including this study’s GIH cohort (Tables S1 and S2). Proposal and approval for databank use was approved by the Data Commission.

### 2.3. Outcome Variables

The main medical outcome variables were the number of diagnoses, type of main or comorbid diagnosis, number of medications, and blood levels of the malnutrition markers vitamin D3 (25-hydroxyvitamin-D, 25-OH-D) and albumin. Main diagnoses (MD) (Table S3) were the indications of being hospitalized and comorbid diagnoses (CD) (Table S4) were the additional diagnoses grouped by body systems (ICD-10 Germ) [26]. The data were part of the medical files. The main dental outcome variables were dental status and intraoral risk factors (IRF), specifically severe bone crest atrophy (SBCA) and masticatory function (MF) [8,12,13] (Table 1). Furthermore, orofacial/neurological risk factors, which represent mental and cognitive impairment, were surveyed as they also impact mastication (Table S1). In current clinical practice, there is no feasible low effort, low cost, in-hospital, bedside method for the objective evaluation of GIH patients’ masticatory assessment. Therefore, this study used the focused kind of MF, which can be recognized objectively from oral examination and dental status. MF was researched by the calculation of two indices: DMF-T [27,28] (Table S1), Eichner Index and the Eichner Score [29] (Table S5), as independent variables. Firstly, as a morbidity measure of natural dentition, the DMF-T Index states the number of decayed, missing and filled natural teeth caused by caries referred to the unit of one tooth (-T) [3,30]. To fit the population of elderly and edentulous GIH patients (Table S6), this study used the adapted version DMF*-T, which includes all destroyed/diseased (D), missing teeth/units (M) and number of fabricated artificial units (F). The adapted DMF*-T Index also considers the impact of artificial teeth and dentures on the mastication and nutrition of GIH patients [27,28] (Table S7). Secondly, the Eichner Index (EI) was used as a clinically suitable index of prosthodontic morbidity. Both indices define the masticatory function to classify the degree of edentulism before treatment compared to the natural dentition [13,29,31]. The EI is based on occlusal contacts between naturally existing teeth in the premolar and molar regions. At least 20 remaining teeth and 8 occluding contact zones were postulated for good mastication [9,11,16]. The average EI was calculated using the Eichner Score with numerical values for each Eichner group on an ordinal scale (A1 = 1 up to C3 = 10), with high EI scores representing insufficient masticatory function (Tables S5 and S6) [15,29,31]. These prosthodontic measures were objective and accessible according to the dental files and constitute the actual sum of dental masticatory function. The medical and dental outcome measures started at baseline after referral to a consultant dentist, which was caused by acute new dental/oral pathological findings.

### 2.4. Data Collection

The in-hospital data administration system for geriatric patients (Geri-DOC) and the medical files provided socio-demographic data and the MDs leading to hospitalization. CD also affects the overall health of in-hospital patients and provides additional informations about the severity and meaning of diseases grouped by body systems. All diagnoses were based on the ICD-10 German modification [26]. Furthermore, routine values were collected by the general medical referral information system (ORBIS). Finally, dental state was assessed by anamnesis, oral examination and questionnaires for the GIH cohort. Due to the severe health condition and mild cognitive impairment of the majority of GIH patients, the questionnaires were filled out by the treating dentist or geriatrician in a patient interview. Additionally, data from the multi-center geriatric databank GiB-DAT included the following clinical, functional and mental measures: the length of stay in hospital counted per day indicates morbidity and outcome of rehabilitation [32], the Barthel Index measures the participation score that defines the capability of daily activities or degree of care dependency [33], the timed Up-and-Go test measures mobility and frailty risk by time to go for a specific distance [34], the mini-mental state examination evaluates cognitive impairment by questionnaires [35], and the Charlson Comorbidity Index scores the mortality to calculate the ten-year survival rate [36] (Table S2).

### 2.5. Statistical Analysis

First, descriptive data were measured. This included age and gender as demographic data, and the following measures of (oral) health status: number of medical diagnoses, need for general and prosthetic dental care, orofacial/neurological risk factors, and intraoral risk factors.

Based on the research question concerning relationships between dental/oral and general health of geriatric patients, the data analysis followed an explorative approach. Accordingly, *t*-tests for independent samples, Mann–Whitney U tests and Spearman’s rank correlations were conducted to calculate correlations and differences between outcome variables. A significance level of *p* < 0.05 was chosen for all statistical analyses. Effect size is shown by Pearson’s correlation coefficient r (*r* < 0.30 = small effect; *r* < 0.50 = medium effect; *r* > 0.50 = large effect). All data were analyzed using Microsoft Excel and IBM SPSS (version 24).

## 3. Results

### 3.1. Descriptive Analysis

Baseline demographic and health status data (Table S7) revealed a mean age of 81.9 years for GIH. The number of medical diagnoses was high (13.86) compared to the average population (Table S7). The need for immediate prosthetic treatment was high (79%) (Table S7). Furthermore, orofacial/ neurological risk factors were found for the entire GIH cohort (Table S7). Almost all GIH patients used removable or full dentures (91%) (Table S2). Additionally, 67% of GIH patients had a renal disorder and suffered from deficiency of vitamin D3 levels (73%) or albumin (86%). The GiB-DAT databank [25] revealed the mean age, the percentage of female patients, the number of diagnoses and the amount of drugs taken, which were almost equal to the single center GIH cohort (Coburg). Referring to the Bavarian rehabilitation population, GIH patients at the Coburg rehabilitation department (mean 733/year) were younger and female, and the number of drugs administrated was smaller [25] (Table S1). Clinical, functional and mental measures emphasize the very high morbidity of the rehabilitation cohort compared to the large geriatric population from the GiB-DAT databank (Table S2).

### 3.2. GiB-DAT Databank of Geriatric Assessment

Compared to the data from the GiB-DAT databank, the rehab department cohort from Coburg was characterized by: (1) a 6 day longer length of stay (2) smaller Barthel Index values at admission, representing a lower capability of daily activities, (3) larger sub-group with lower mobility and higher independency, as measured by the timed Up-and-Go test, and (4) reduced mini-mental state scores indicating dementia. The study cohort’s patient selection underlines the extremely high morbidity consisting of both general and dental morbidity signs, such as SBCA and impaired MF (Tables S1 and S2).

### 3.3. Inferential Analysis

The inferential analysis before immediate prosthetic treatment was essential for the status at referral of GIH. The influence of the IRF inflammation and mucogingival impairment (MGI) on the health condition revealed statistically significant correlations. This was validated by a significant difference in patients’ number of diagnoses depending on the occurrence of these IRFs (Table 1).

For dental status, a significant difference between masticatory function, measured by EI scores, between patients with and without intraoral morbidity of jaw bone, indicated by SBCA, was found through a Mann–Whitney U test. The masticatory function of GIH patients with SBCA was significantly lower compared to patients without SBCA (Table 2).

The levels of 25-OH-D (vitamin D3) of GIH patients also differed depending on the occurrence of SBCA. Albumin levels, on the other hand, were not associated with SBCA (Table 3).

Mann–Whitney U tests showed 25-OH-D levels to be 2.5 times lower in patients with a main diagnosis of CNS disorders than in those without (*p* = 0.011; *r* = 0.45). For patients with gastro-intestinal disease (GID), on the other hand, 25-OH-D levels were significantly higher with medium effect sizes compared to patients without (*p* = 0.028; r = 0.40) (Table 4). A 25-OH-D-level of 20.0–29.00 ng/mL equaled mild to moderate insufficiency, and values < 20 ng/mL indicated equal deficiency. The groups with immobility and pulmonal disease as MDs were thus classified as vitamin D3 deficient and the CNS group as vitamin D3 insufficient.

Similarly, albumin values differed significantly with medium effect sizes between GIH patients with and without MDs, such as CNS disorder, immobility, GID and pulmonal disease, after being hospitalized. Patients with CNS disorders and immobility had higher albumin values than patients without, whereas patients with GID and pulmonal disorder had lower albumin values than those without (Table 4). Other MDs did not yield significant correlations (Table S3).

For comorbid diagnoses, vitamin D3 differed significantly with medium effect between GIH patients with and without cardiovascular disease. Patients with cardiovascular disease had lower 25-OH-D-levels than patients without (Table 5). Albumin values also differed significantly with weak effect sizes between GIH patients with or without diabetes mellitus (Table S4).

A significant negative correlation with a weak effect size between albumin values and the number of CDs could also be demonstrated by a Spearman’s rank correlation. Therefore, lower albumin levels were associated with a higher number of CDs in GIH patients. For 25-OH-D levels, no such correlation could be detected (Table 6).

## 4. Discussion

In previous literature, the significant correlation between masticatory function and EI has been shown for existing natural teeth or fixed protheses but had not been researched before for removable protheses of GIH patients [13]. Finding significant associations in a cohort with almost exclusively removable prostheses, this study thus importantly adds to existing research. Therefore, MF is an appropriate objective measure and relevant for clinical application and further research. The key results of this retrospective analysis showed high morbidity, significant intraoral risk factors, impaired masticatory function and significant malnutrition markers for GIH patients. This observation can be recognized as indicating predictors for possible health complications and higher mortality. These predictors are associated with progressive edentulism and consequently with SBCA causing impaired masticatory function, which in turn facilitates insufficient nutrition [37] and malnutrition [17]. As previously mentioned in this paper, some main diagnoses significantly correlated with vitamin D3 and albumin levels as malnutrition markers. This correlation may be associated particularly with CNS disorders, specifically MCI [17], as a precondition of insufficient food intake caused by disregard for the oral condition and missing sensation of hunger and impaired neuro-muscular function. CNS disorders seem to be malnutrition risks indicated by impaired cognition and immobility and might be associated with reduced oxygen supply [22,23,38]. Patients with other disorders, on the other hand, had lower vitamin D3 and albumin values, as renal insufficiency and albumin metabolism may be related to impaired oxygen and nutrition supply of this extraordinary old GIH cohort. The total number of diagnoses, which is associated with the deficiency of albumin levels, represents the overall disease burden. Deficient vitamin D3 levels and albumin levels are not only risk markers for malnutrition but also prevalent in kidney diseases, which constitute a high risk for general health [39]. According to the Kidney International Executive Summary 2017 [40], recommended values of vitamin D3 for the general population are also recommended for patients with chronic kidney disease (G1–G5D). Previous literature has further stated that vitamin D3 levels are protective against arteriosclerotic disease, MCI, dementia and Alzheimer’s disease, although these so-called pleiotropic effects are still controversial [3,41,42]. This effect may be caused by cerebrovascular dysfunction leading to dysfunctional mastication and thus to malnutrition induced by impaired oxygen supply, neuromuscular control, cognition and selfcare. As a longitudinal study found that reduced chewing ability influenced cognitive impairment in older adults [38], tooth loss is also an important risk factor for cognitive impairment and dementia itself [22]. The association between vitamin D3 deficiency and SBCA, as found in this study, can be derived from the impact of vitamin D3 on calcium and bone metabolism. When vitamin D3 levels are reduced, muscle and bone health and capability can be impaired, and immune system and wound healing can be harmed [39,40]. Thus, osteoporosis, bone softening and diabetes mellitus can arise and in consequence aggravate SBCA. The present study also showed that an important part of vitamin D3 and albumin deficiency may be caused by malnutrition and impaired MF in the first place. Malnutrition may be assumed to be a consequence and predictor of high general [18] and dental [19] morbidity, which is significant for the GIH cohort. Nutrition-related diseases are chronic diseases [3] that last for decades and cannot be improved or healed easily or efficiently but can be improved. Furthermore, they can emphasize and cause morbidity compression [2], meaning that they are lifestyle-dependent and self-aggravating and cumulate with rising age and decreasing intraoral condition [2]. As a consequence, nutrition-related diseases lead to cumulative healthcare costs by expensive health care and high life expectancy in old age [18]. Currently, in Germany, the in-patient dental assessment is not part of the hospital routine and not covered by public health systems. Dental care is relatively low in cost and is only covered for patients in rehabilitation departments. Incorporating this early diagnosis, especially the dental assessment, into clinical routine could thus lead to significant cost savings. The results of this study thus underline the importance of dental care and in-hospital cooperation of physicians and dentists worldwide [43], and recommendations for policymakers may become realizable.

This study shows some limitations, first the small sample size. Further, single center recruitment and convenience sampling as sampling methods provide potential biases. Therefore, further confirmatory research studies with larger sample sizes and studies after PT should be considered for future research. Internal and external validity may generally be considered as weaknesses of retrospective cohort studies. The present study, however, addresses this weakness by using scientifically established indices as outcome measures to improve internal validity and additionally compares the collected data with the large Gib-DAT databank to improve external validity. Multiple statistically significant findings as well as great clinical relevance further underline the scientific contribution of this research.

## 5. Conclusions

This study revealed that impaired masticatory function and malnutrition might become predictors for impaired general health. Malnutrition can be caused by reduced masticatory function, high general morbidity and intraoral risk factors, particularly SBCA. Furthermore, vitamin D3 and albumin levels were possible key markers of malnutrition. However, GiB-DAT data support our results as representative and validate the novelty and relevance of this dental assessment for diagnosis and treatment planning for this very vulnerable population [24,25]. The results thus emphasize the early preventive and efficient diagnostic methods. Since these methods are effective and achievable in clinical routine, they can be recommended for both geriatric and non-geriatric in- and out-patients.

## Figures and Tables

**Table 1:** Number of medical diagnoses of GIH patients depending on the occurrence of IRFs (*t*-test for independent samples).

Intraoral Risk Factors	Affected	No. of Diagnoses		*p*	95%-CI	*r*
		M	SD			
Inflammation	yes (n = 61)	14.54	4.85	0.024 *	[−5.10; −0.38]	0.25
	no (n = 20)	11.80	3.75			
SBCA	yes (n = 47)	13.66	4.88	0.650	[−1.64; −2.62]	
	no (n = 34)	14.15	4.66			
Oral Pain	yes (n = 42)	14.29	5.19	0.505	[−2.81; 1.39]	
	no (n = 38)	13.58	4.12			
MGI	yes (n = 4)	8.75	1.26	0.026 *	[0.67; 10.09]	0.25
	no (n = 77)	14.13	4.70			

Notes: CI = confidence interval. SBCA = severe bone crest atrophy. MGI = mucogingival impairment. * 2-sided significance.

**Table 2:** Eichner scores of GIH patients with/without SBCA and weight loss (Mann–Whitney U Test).

		EI
Disease	Affected	*p* (*r*)
Weight loss	yes (n = 31)	0.799
	no (n = 49/50)	
SBCA	yes (n = 47)	0.000 (0.46)
	no (n = 33/34)	

Notes: EI = Eichner Index.

**Table 3:** Correlation between SBCA and 25-OH-D levels and albumin levels of GIH patients (*t*-test for independent samples).

Intraoral Risk Factors	Affected	25-OH-D-[ng/mL]		*p*	*r*
		M	SD		
SBCA	yes (n = 18) no (n = 12)	25.47 16.19	13.30 10.10	0.050	0.36
	**Affected**	**Albumin [g/L]**		***p***	***r***
		**M**	**SD**		
SBCA	yes (n = 32) no (n = 24)	33.84 34.49	5.05 4.81	0.630	0.07

Notes: SBCA = severe bone crest atrophy.

**Table 4:** Levels of vitamin D3 and albumin of GIH patients differ depending on the occurrence of single main diagnoses (Mann–Whitney U tests) [excerpt] and Table S3 (full table).

Main Diagnoses	Affected	25-OH-D-[ng/mL]		*p*	*r*
		M	SD		
CNS disorders *	yes (n = 4) no (n = 26)	8.70 23.77	4.53 12.49	0.011	0.45
Immobility	yes (n = 1) no (n = 29)	17.70 21.90	- 13.00	0.867	-
Gastro-intestinal disease	yes (n = 9) no (n = 21)	29.62 18.39	13.32 11.26	0.028	0.40
Pulmonal disease	yes (n = 4) no (n = 26)	15.35 22.74	8.21 13.20	0.328	-
**Main Diagnoses**	**Affected**	**Albumin [g/L]**		***p***	***r***
		**M**	**SD**		
CNS disorders *	yes (n = 9) no (n = 47)	37.54 33.47	2.73 4.99	0.013	0.33
Immobility	yes (n = 5) no (n = 51)	39.20 33.62	2.57 4.82	0.013	0.33
Gastro-intestinal disease	yes (n = 11) no (n = 45)	31.33 34.80	5.03 4.69	0.023	0.30
Pulmonal disease	yes (n = 13) no (n = 43)	30.96 35.08	4.18 4.76	0.006	0.36

Notes: CNS = central nervous system, * If N > 30, the asymptotic significance, and if N ≤ 30, the exact significance is indicated.

**Table 5:** Levels of vitamin D3 and albumin of GIH patients depending on the occurrence of single comorbid diagnoses (Mann–Whitney U tests) [excerpt] and Table S4 (full table).

Comorbid Diagnoses	Affected	25-OH-D-[ng/mL]		*p*	*r*
		M	SD		
Cardiovascular disease	yes (n = 26) no (n = 4)	19.98 33.28	12.33 10.57	0.031	0.39
Diabetes mellitus	yes (n = 3) no (n = 27)	16.47	18.56	0.350	-
		22.34	12.35		
Chronic pain	yes (n = 9) no (n = 21)	26.70 19.64	14.46 11.75	0.209	-
**Comorbid Diagnoses**	**Affected**	**Albumin [g/L]**		***p***	***r***
		**M**	**SD**		
Cardiovascular disease	yes (n = 50) no (n = 6)	34.31 32.55	4.64 7.18	0.633	-
Diabetes mellitus	yes (n = 2) no (n = 54)	26.35 34.41	4.60 4.72	0.042	0.27
Chronic pain	yes (n = 16) no (n = 40)	32.24 34.87	4.64 4.87	0.042	0.27

If N > 30, the asymptotic significance, and if N ≤ 30, the exact significance is indicated.

**Table 6:** Correlation between albumin levels and the number of comorbid diagnoses (CDs) (Spearman’s rank correlations).

	25-OH-D	Albumin
	*p* (*r*)	*p* (*r*)
**Number of CDs**	0.277	0.033 (−0.29)

## Data Availability

The data presented in this publication are available on request from the corresponding author. The data are not publicly available due to privacy restrictions.

## Supplementary Materials

The following supporting information can be downloaded at: https://www.mdpi.com/article/10.3390/dj13080334/s1, Table S1: Comparison between federate state population and single hospital GIH cohort (Coburg), demographic data and general health status. Table S2: Comparison between single GIH cohort (Coburg) and total federate state rehab population: Demographic, geriatric and mental measures. Table S3: Levels of vitamin D3 and albumin of GIH patients differ depending on the occurrence of several main diagnoses (Mann-Whitney-U tests). Table S4: Levels of vitamin D3 and albumin of GIH patients differ depending on the occurrence of several comorbid diagnoses grouped by body system (Mann-Whitney-U tests). Table S5: Schematic representation of the Eichner Index (EI) [29]. Table S6: State of denture before IPT. Table S7: Baseline demographic data and health status for GIH.
