# Supplementary material for: Dental, Oral and General Health of Geriatric In-Hospital Patients Before Immediate Prosthetic Treatment: A Retrospective Cohort Study
Source: Dent J (Basel). 2025 Jul 22;13(8):334. doi: 10.3390/dj13080334 (PMC12385135; doi:10.3390/dj13080334)
Supplement: Supplementary file 1 [file dentistry-13-00334-s001.zip › dentistry-3680207-supplementary.pdf]

dentistry journal

MDPI

Supplementary Materials:.

Table S1. Comparison between federate state population and single hospital GIH cohort (Coburg), demographic data and general health status.

	Bavaria			Coburg		
	**2015**	**2016**	**2017**	**2015**	**2016**	**2017**
**Mean age**	81.49	81.40	81.67	80.93	80.59	81.35
**Percentage of women**	66.41	66.54	67.04	61.84	60.26	60.00
**No. of medical diagnoses (main and comorbid)**	13.27	12.89	13.01	8.14	8.46	3.41
**No. of medications**	8.80	8.90	9.01	--	--	--
**No. of hospitals**	55	58	56	1	1	1
**No. of patients**	34960	37054	37531	718	760	720

Notes*:* This data does not originate from this study, but from the GiB-Dat databank and serves as a comparison to our results.

Table S2. Comparison between single GIH cohort (Coburg) and total federate state rehab population: Demographic, geriatric and mental measures.

	**Coburg GIH rehab department**	**Total of Rehab departments in Bavaria 2015 – 2017 without patients from Coburg**
**Number of cases**	61	107,347
**Age (years)**	82.02	81.53
**Proportion of women [%]**	69.3	66.79
**Length of stay [days]**	27.93	21.79
**Barthel index at admission**	35.67	46.54
**Barthel index at discharge**	58.67	68.67
**MMSE**	22.39	24.42
**TUG at admission [%]** [a]	33.33 - 35.29 - 31.37	55.74 - 29.28 - 14.98
**TUG at discharge [%]** [a]	84.09 - 11.36 - 4.55	79.77 - 15.18 - 5.05
**CCI**	4.46	4.69

Notes: This data does not originate from this study but from the GiB-DAT databank and serves as a comparison to our results. Only 61 out of our study group (n=81) could be calculated caused by missing data. MMSE = mini mental state examination. TUG = timed up and go test. CCI = Charlson-Comorbidity- Index [a] independently ambulatory - ambulatory with assistance - not ambulatory.

Table S3. Levels of vitamin D3 and albumin of GIH patients differ depending on the occurrence of several **main** diagnoses (Mann-Whitney-U tests).

**n**	**25-OH-D- [ng/ml]**					**Albumin [g/l]**				
**Main Diagnose**	**Affected**		**Not affected**			**Affected**		**Not affected**		
	**N**	**M**	**N**	**M**	***p*** **(*r*)**	**N**	**M**	**N**	**M**	***p*** **(*r*)**
**F**	11	24.22	19	20.33	0.328	24	34.18	32	34.08	0.974
**CVD**	9	18.14	21	23.30	0.397	16	34.11	40	34.13	0.964

**DM**	2	31.85	28	21.04	0.193	3	28.63	53	34.43	0.184
**N**	7	24.71	23	20.86	0.413	8	31.36	48	34.58	0.083
**CNS**	4	8.70	26	23.77	0.011 (.45)	9	37.54	47	33.47	0.013 (.33)
**M**	0	-	30	21.76	-	0	-	56	34.12	-
**Bl**	0	-	30	21.76	-	0	-	56	34.12	-
**Fr**	7	15.83	23	23.56	0.207	7	36.71	49	33.75	0.119
**I**	1	17.70	29	21.90	0.867	5	39.20	51	33.62	0.013 (.33)
**GID**	9	29.62	21	18.39	0.028 (.40)	11	31.33	45	34.80	0.023 (.30)
**P**	4	15.35	26	22.74	0.328	13	30.96	43	35.08	0.006 (.36)
**Ps**	0	-	30	21.76	-	2	37.40	54	34.00	0.251
**CP**	2	10.55	28	22.56	0.166	0	-	56	34.12	-
**C**	2	43.55	28	20.20	0.055	2	33.60	54	34.14	0.842
**S1**	4	27.33	26	20.90	0.391	6	34.40	50	34.09	0.979

Notes. MD = Main diagnosis. F = Fracture, Trauma, Rupture, etc. CVD = Cardiovascular. DM = Diabetes Mellitus. N = Nephrological. M = Metabolism. Bl = Blood. Fr = Frailty. I = Immobility. GID = Gastro-intestinal. P = Pulmonal. Ps = Psychological. CP = Chronic Pain. C = Cancer. S1 = Surgery. * If N > 30 the asymptotic significance and if N ≤ 30 the exact significance was indicated.

Table S4. Levels of vitamin D3 and albumin of GIH patients differ depending on the occurrence of several **comorbid** diagnoses grouped by body system (Mann-Whitney-U tests).

	**25-OH-D- [ng/ml]**					**Albumin [g/l]**				
**Comorbid Diagnose**	**Affected**		**Not affected**			**Affected**		**Not affected**		
	**N**	**M**	**N**	**M**	***p* (*r*)**	**N**	**M**	**N**	**M**	***p* (*r*)**
**CVD**	26	19.98	4	33.28	0.031 (.39)	50	34.31	6	32.55	0.633
**GID**	15	24.48	15	19.03	0.325	26	34.10	30	34.14	0.882
**En**	21	22.45	9	20.14	0.894	33	33.80	23	34.59	0.677
**DM**	3	16.47	22	34.00	0.350	2	26.35	54	34.41	0.042 (.27)
**Ly**	1	24.00	29	21.68	0.800	1	31.50	55	34.17	0.421
**Mu**	2	12.90	28	22.39	0.414	1	41.40	55	33.99	0.146
**Ne**	23	19.97	7	27.63	0.077	43	34.69	13	32.25	0.171
**Re**	21	20.20	9	25.38	0.397	36	33.85	20	34.61	0.614
**Rp**	4	13.15	26	23.08	0.082	7	33.09	49	34.27	0.488
**Rs**	10	16.05	20	24.61	0.100	20	32.65	36	34.94	0.070
**Sk**	23	23.20	7	17.00	0.360	46	33.90	10	35.15	0.404
**Im**	0	-	30	21.76	-	0	-	56	34.12	-
**Bl**	15	20.93	15	22.58	0.775	18	32.76	38	34.77	0.191
**CP**	9	26.70	21	19.64	0.209	16	32.24	40	34.87	0.042 (.27)

Notes: BS = Body System. CVD = Cardiovascular. GID = Gastro-intestinal. En = Endocrine. DM = Diabetes mellitus. Ly = Lymphatic. Mu = Muscular. Ne = Nervous; Re = Renal. Rp = Reproductive.

Rs = Respiratory. Sk = Skeletal. Im = Immune. Bl = Blood. CP = Chronic pain.* If N > 30 the asymptotic significance and if N ≤ 30 the exact significance is indicated.

Table S5. Schematic representation of the Eichner Index (EI) [29.]

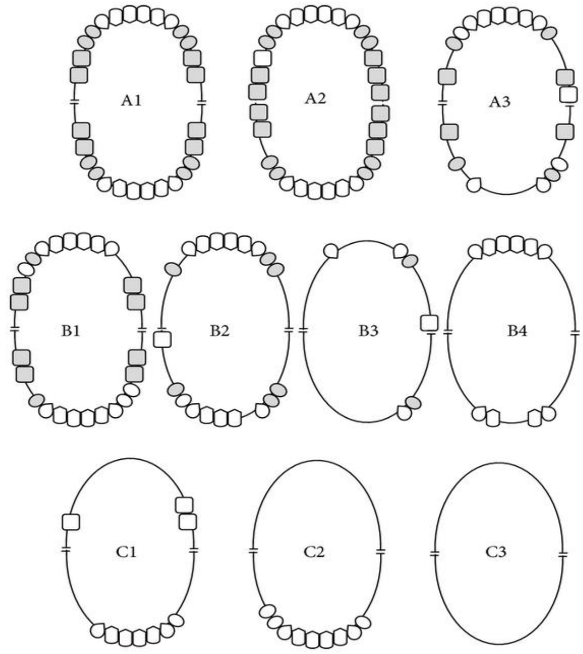

Category A contains all four occlusal support zones
A1 – complete dentition
A2 – missing teeth in one arch
A3 – missing teeth in both arches

Category B contains one to three occlusal support zones or contacts in the anterior area only
B1 – three occlusal support zones
B2 – two occlusal support zones
B3 – one occlusal support zone
B4 – contacts in the anterior area only

Category C has no occlusal support zones at all
C1 – teeth in both arches
C2 – teeth in one arch
C3 –

Notes*:* For calculations, the EI's were scaled as follows: A1 = 1; A2 = 2; A3 = 3; B1 = 4; B2 = 5; B3 = 6; B4 = 7; C1 = 8; C2 = 9; C3 = 10.

Table S6. State of denture before IPT.

	**GIH (n = 81)**
**Patients with removable dentures (RD) [%]**	91
**Patients with fixed dentures (FD) or natural dentition (ND) [%]**	9

Table S7. Baseline demographic data and health status for GIH .

**Demographic and health status**		**GIH (n = 81)**
**Age**		81.9
**Gender**	Male [%]	38
	Female [%]	62
**(Oral) health status**		
No. of medical diagnoses [M ± SD]		13.86 ± 4.73
Need of general dental care [%]		41

Need of removable prosthetic therapy [%]	74
Need of fixed prosthetic therapy [%]	5
Orofacial/ neurological risk factors [%]:	
CNS disorders	44
Dementia	31
Orofacial Disorders	10
Intraoral risk factors [%]:	
Inflammation	75
SBCA	58
Oral Pain	53
MGI	5

	**GIH (n = 81)**
**Average DMF*-T**	25.58

Notes: CNS = central nervous system. SBCA = severe bone crest atrophy. MGI = Mucogingival impairment. DMF*-T = adapted version of the measure of dental morbidity DMF-T. 99% of the GIH patients are over 60 years old.
